# Technologies for Interoperable Internet of Medical Things Platforms to Manage Medical Emergencies in Home and Prehospital Care: Scoping Review

**DOI:** 10.2196/54470

**Published:** 2025-01-23

**Authors:** Mattias Seth, Hoor Jalo, Åsa Högstedt, Otto Medin, Bengt Arne Sjöqvist, Stefan Candefjord

**Affiliations:** 1 Department of Electrical Engineering Chalmers University of Technology Gothenburg Sweden; 2 Prehospen – Centre for Prehospital Research, Faculty of Caring Science, Work Life and Social Welfare University of Borås Borås Sweden; 3 InterSystems Corp Stockholm Sweden

**Keywords:** Internet of Medical Things, enabling technologies, standards, cross-domain interoperability, scoping review, technology, medical emergency, internet, prehospital care, gerontology, global population, chronic disease, multimorbidity, health care system, home-based care, innovation, digital health, health informatics, telehealth, artificial intelligence

## Abstract

**Background:**

The aging global population and the rising prevalence of chronic disease and multimorbidity have strained health care systems, driving the need for expanded health care resources. Transitioning to home-based care (HBC) may offer a sustainable solution, supported by technological innovations such as Internet of Medical Things (IoMT) platforms. However, the full potential of IoMT platforms to streamline health care delivery is often limited by interoperability challenges that hinder communication and pose risks to patient safety. Gaining more knowledge about addressing higher levels of interoperability issues is essential to unlock the full potential of IoMT platforms.

**Objective:**

This scoping review aims to summarize best practices and technologies to overcome interoperability issues in IoMT platform development for prehospital care and HBC.

**Methods:**

This review adheres to a protocol published in 2022. Our literature search followed a dual search strategy and was conducted up to August 2023 across 6 electronic databases: IEEE Xplore, PubMed, Scopus, ACM Digital Library, Sage Journals, and ScienceDirect. After the title, abstract, and full-text screening performed by 2 reviewers, 158 articles were selected for inclusion. To answer our 2 research questions, we used 2 models defined in the protocol: a 6-level interoperability model and a 5-level IoMT reference model. Data extraction and synthesis were conducted through thematic analysis using Dedoose. The findings, including commonly used technologies and standards, are presented through narrative descriptions and graphical representations.

**Results:**

The primary technologies and standards reported for interoperable IoMT platforms in prehospital care and HBC included cloud computing (19/30, 63%), representational state transfer application programming interfaces (REST APIs; 17/30, 57%), Wi-Fi (17/30, 57%), gateways (15/30, 50%), and JSON (14/30, 47%). Message queuing telemetry transport (MQTT; 7/30, 23%) and WebSocket (7/30, 23%) were commonly used for real-time emergency alerts, while fog and edge computing were often combined with cloud computing for enhanced processing power and reduced latencies. By contrast, technologies associated with higher interoperability levels, such as blockchain (2/30, 7%), Kubernetes (3/30, 10%), and openEHR (2/30, 7%), were less frequently reported, indicating a focus on lower level of interoperability in most of the included studies (17/30, 57%).

**Conclusions:**

IoMT platforms that support higher levels of interoperability have the potential to deliver personalized patient care, enhance overall patient experience, enable early disease detection, and minimize time delays. However, our findings highlight a prevailing emphasis on lower levels of interoperability within the IoMT research community. While blockchain, microservices, Docker, and openEHR are described as suitable solutions in the literature, these technologies seem to be seldom used in IoMT platforms for prehospital care and HBC. Recognizing the evident benefit of cross-domain interoperability, we advocate a stronger focus on collaborative initiatives and technologies to achieve higher levels of interoperability.

**International Registered Report Identifier (IRRID):**

RR2-10.2196/40243

## Introduction

### Background

The aging world population and an increased prevalence of chronic conditions and multimorbidity have placed greater pressure on health care systems and professionals [1]. In Europe, over 50 million people have at least 1 chronic disease [2]; and in the United States, it is estimated that the number of people aged 50 years and older with at least 1 chronic disease will increase by 100%, from 71 million in 2020 to 142 million by 2050 [3]. The incidence of life-threatening falls among older adults (aged ≥70 y) continues to rise globally [4]; together with other age-related medical emergencies, such as stroke [5], these events claim millions of lives each year [5,6]. The notable changes in global demographics, combined with the prevalence of frailty among older adults, underscore the importance of expanding the human resources available in the public health sector. Nevertheless, due to economic and occupational constraints, this may not be a feasible solution [7]. To alleviate the pressure on health care systems, more sustainable initiatives need to be implemented. One potential solution often discussed in the literature is the transition to home-based care (HBC) [8,9]. HBC covers a wide continuum of care and involves delivering increasingly complex health care services to individuals in their own residences, allowing them to maintain their independence as an alternative to relying on residential, long-term, or institutional nursing care [10,11]. This transition is often supported by older adults because the majority choose to remain in their own homes for as long as possible [12,13].

**Figure 1:**
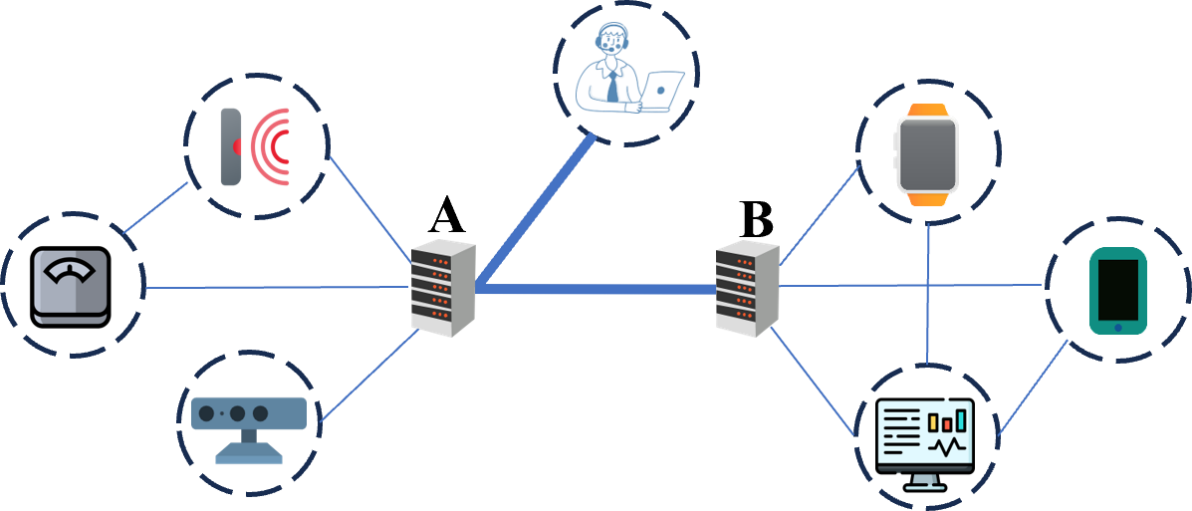
In this study, the Internet of Medical Things (IoMT) was defined as a network of interconnected medical devices and health care systems that use the internet to collect, transmit, and exchange health care data. The figure illustrates device-to-device (thin lines), device-to-system (thin lines), and system-to-system (thick lines) communication, with 2 IoMT platforms (A and B) interacting with each other.

To support the transitioning to HBC, there is a growing demand for new technological innovations and collaborative initiatives [14]. This often involves modifications of peoples’ homes and the use of medical equipment that not only facilitates long-term health monitoring but also enables the management of medical emergencies that demand rapid medical attention [15-17]. This is particularly important in the case of older adults (aged ≥65 y) because their inability to manually initiate an emergency alarm after a sudden deterioration in physical condition (eg, acute stroke or hip dislocation after a fall) may lead to long-term consequences and increased mortality [15]. Studies have shown that Internet of Medical Things (IoMT; Figure 1) platforms have the potential to support the transitioning to HBC by streamlining workflows, reducing costs and time delays, and improving patient well-being [18-20]. While previous research has demonstrated the usefulness of stand-alone platforms for the monitoring of chronic conditions and detection of abnormalities, there has been limited focus on achieving higher levels of interoperability within IoMT [21-24]. Although stand-alone platforms may be feasible in certain situations, the lack of interoperability with external systems such as electronic health records (EHRs), emergency medical services, and public safety answering points (PSAPs) may lead to data becoming trapped within silos, potentially resulting in delayed information transfer, suboptimal decision-making, and patient harm [7,21,25,26]; for example, a study conducted by Magrabi et al [27] showed that approximately 20% of reported patient safety hazards were linked to deficient information transfer. Furthermore, Hyvämäki et al [26] showed that inadequate documentation and use of information in HBC plays a significant role in interorganizational health information exchange–related incidents, resulting in, for example, delayed care and patient harm.

At Chalmers University of Technology in Gothenburg, Sweden, the Care@Distance research group is active in the field of remote and prehospital digital health. The group is dedicated to improving health care delivery through interoperable cutting-edge solutions, encompassing clinical decision support systems, artificial intelligence (AI), modern IT, and innovative user interactions. While interoperability offers numerous advantages, we acknowledge that current medical data still contain nonstandard elements [28,29], and vendors continue to use their own proprietary solutions [29]; for example, most inpatient EHRs include over 5000 variables [28,29], making it difficult to ensure a shared understanding of medical concepts across domains and organizations [29]. One system may code “A fall on and from stairs and steps” as W10 in the *International Classification of Diseases, Tenth Revision, Clinical Modification* (*ICD-10-CM*), while another system uses Systematized Nomenclature of Medicine–Clinical Terms (SNOMED CT) code 900000000000448009 for “Fall on stairs.” Establishing a shared understanding of these 2 terms requires a translation [21]. As the number of unique systems and standards increases, the need for separate translations also increases, especially considering that SNOMED CT includes over 340,000 medical concepts (clinical findings, procedures, substances, etc) [29]. Due to these complex translation processes within health care, involving various file formats (eg, text, video, images, and audio), communication protocols, and semantics, over 80% of all medical data tend to be overlooked or discarded [19]. This not only hampers communication between systems but also limits the use of AI, international cooperation, and research [29]. As the technological landscape expands, navigating among the available technologies and standards is becoming increasingly challenging. Hence, in this study, we summarize existing knowledge and best practices to overcome interoperability issues in IoMT platform development to manage medical emergencies within HBC and prehospital care settings.

### Objectives

This scoping review aims to summarize and map the enabling technologies that can be used to develop interoperable platforms to manage medical emergencies in HBC and prehospital care. We have proceeded from a 6-level interoperability model comprising device, network, syntactical, semantic, cross-platform, and cross-domain interoperability and a 5-level IoMT reference model comprising perception, transport, processing, application, and business layers [11]. These models provide adequate granularity and context to be applied within the context of IoMT [10]. Relevant actors and domains within the IoMT domain include homes, sensor providers, emergency medical services, PSAPs, social security services, and hospitals. The aim is to describe technologies and their use in an accessible way, enabling cross-disciplinary discussion between clinicians and engineers. This scoping review can potentially serve as a guide for software developers, clinicians, and other practitioners aiming to develop interoperable IoMT platforms.

## Methods

### Overview

**Figure 2:**
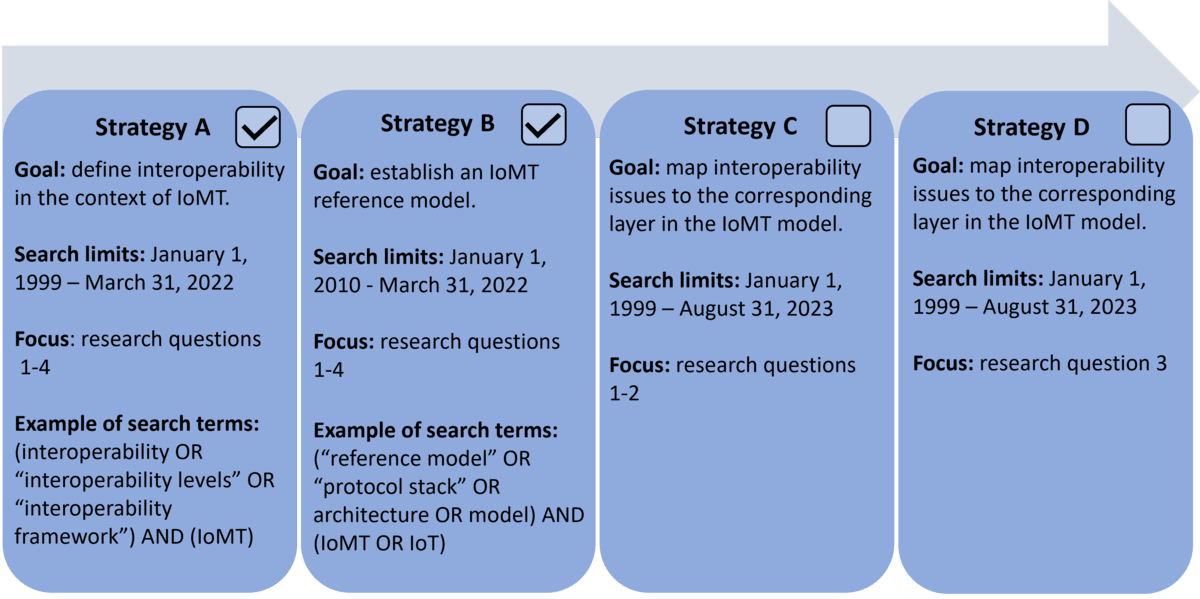
This scoping review used a 4-strategy approach to streamline the search process. Strategies A and B were conducted as part of the research protocol [11], while strategies C and D are addressed in this scoping review. HBC: home-based care; IoMT: Internet of Medical Things.

This scoping review follows the PRISMA-ScR (Preferred Reporting Items for Systematic Reviews and Meta-Analyses extension for Scoping Reviews) guidelines for scoping reviews (refer to Multimedia Appendix 1 for the PRISMA-ScR checklist) [30] and adheres to the methodology outlined in the research protocol [11]. Given the broad spectrum of research questions addressed in this scoping review, the methodology includes 4 distinct search strategies, referred to as strategies A to D in the research protocol [11]. The relationships between these search strategies are illustrated in Figure 2.

This approach allows each research question to be addressed more systematically by separating the search terms, search periods, and goals within each search strategy. Search strategies A and B were completed with the research protocol [11] (Figure 2) and addressed the following research questions:

1. What are the current challenges of developing a real-time IoMT platform for managing medical emergencies such as falls?
2. What is interoperability? How can it be defined in the context of IoMT?
3. What types of models are used to visualize the different layers of interoperability? When talking about medical devices in an IoMT setting, which model is preferable and why?
4. Which reference model with corresponding protocols can best describe and define the structure of key aspects of the information being managed in a real-time IoMT system? How is the model being used today?

These 4 research questions were addressed in the research protocol [11], resulting in the definition of a 6-level interoperability model and an IoMT reference model to be used as reference materials in this scoping review. On the basis of these definitions, this scoping review will proceed with search strategies C and D (Figure 2), focusing on answering the following research questions:

1. Have any studies examined which current technologies are associated with the layers in the IoMT reference model, comprising device, network, syntactical, semantic, cross-platform, and cross-domain interoperability, and how these are being used to fulfill the set of rules defined by each layer? If so, what are the results?
2. How can interoperability solutions be mapped to the layers in the interoperability model?
3. What recommendations regarding enabling technologies can be given to clinicians and practitioners who want to develop IoMT platforms that can aggregate, store, and process data from relevant actors in prehospital care and HBC settings?

Research questions 1 and 2 are addressed in search strategy C, and research question 3 is addressed in search strategy D in this review.

### Search and Screening Process

Six electronic databases were used in this study: IEEE Xplore, PubMed, Scopus, ACM Digital Library, Sage Journals, and ScienceDirect [11]. In the published protocol, we had proposed using the Google Scholar database, but we replaced it with the ACM Digital Library in this review to ensure higher precision in search results [31,32] (refer to Multimedia Appendix 2 for the search terms used for search strategies C and D). The retrieved articles were assessed for inclusion based on predefined eligibility criteria and underwent a 2-step screening process using the web application Rayyan (Rayyan Systems Inc) [33]. The screening was performed by HJ and MS, and any disagreements were resolved through discussion between them [11].

### Eligibility Criteria

The eligibility criteria were originally defined in the research protocol [11]. However, a minor adjustment was necessary to streamline the screening process and enhance clarity (Textbox 1). The last inclusion criterion was updated to emphasize a stronger focus on technologies that address interoperability issues.

**Eligibility criteria.**

**Inclusion criteria**

- Published peer-reviewed journals and conference papers
- Written in English
- Published between January 1,1999, and August 31, 2023
- Studies describing or reporting the development or design of Internet of Medical Things (IoMT) systems with a focus on technology
- Studies reporting challenges and barriers to integrating IoMT platforms into prehospital care or home-based care settings with a focus on technology
- Studies describing the enabling technologies that can be used to solve interoperability issues in IoMT platform development

**Exclusion criteria**

- Full-text articles that were unavailable or not written in English
- Conference abstracts, book reviews, commentaries, and editorial articles
- Studies focusing on hardware, project management processes, or regulatory compliance
- Studies reporting on the design or development of IoT applications with no focus on health data (eg, Industry 4.0, including the automotive, food, and manufacturing industries)
- Studies describing the design or development of machine learning methods to achieve interoperability

### Data Extraction

In search strategy C, data were extracted to map interoperability solutions to their respective interoperability models, categorizing them based on the specific level of interoperability addressed. The mapping was based on the 6-level interoperability model and the 5-level IoMT reference model established in the research protocol [11]. Similar mappings that could be found in the literature acted as a reference and, combined with the expertise of our research group, were used to validate the mappings.

In search strategy D, previous efforts in IoMT platform development for prehospital care and HBC were examined. A thematic analysis approach was used to identify and summarize the technologies and standards used in these development processes (Textbox 2). This analysis was performed by 1 reviewer (MS) using Microsoft Excel (version 2403) and the web application Dedoose (version 9.0.107; SocioCultural Research Consultants LLC). The thematic analysis was based on the framework outlined by Braun and Clarke [34]. The framework was adapted to this study and included five steps:

1. Reading through the literature and recording valuable aspects of the data, including the technologies and standards used
2. Organizing data into meaningful groups and creating codes relevant to the research questions (each technology was assigned a code, represented by a short descriptive summary of its intended use; Textbox 2)
3. Collating codes and assigning different keywords to them (each code could be assigned multiple keywords; the keywords were used to facilitate thematic analysis, aiming to conceptualize the functionality of each level in the interoperability model)
4. Assigning a nonoverlapping theme to each technology based on the codes and keywords (technologies with similar codes and keywords were assigned the same theme; a theme was defined as one of the following interoperability levels: device, network, syntactical, semantic, cross-platform, or cross-domain interoperability)
5. Compiling the thematic insights into a coherent review

**An example of the thematic analysis process. The applicability of the mapping was confirmed by our research group through tests with multiple articles [35].**

**Blockchain technology mapped to cross-domain interoperability**

- Technology: blockchain
- Description in literature: “integration of blockchain into healthcare applications, including all aspects of privacy, validity of safety, and access to patient and electronic health records”
- Code assigned: blockchain can be used to preserve privacy and integrity of health data
- Keywords: security, data privacy, and integrity
- Assigned theme: cross-domain interoperability

From the thematic analysis, a list of commonly used technologies was compiled. Each technology was accompanied by a descriptive overview of its application area and function, along with a mapping to one of the levels in the interoperability model defined in the research protocol [11]. For each study, the main areas of interest were identified, such as citation details (eg, author, year of publication, and country of origin) and key study characteristics (eg, type of IoMT platform, platform requirements, interoperability challenges, and the technologies and standards used to achieve different levels of interoperability). The results were summarized using narrative descriptions as well as figures and tables. The data extraction process ensured that sufficient data were gathered to answer each research question.

## Results

### Study Selection

**Figure 3:**
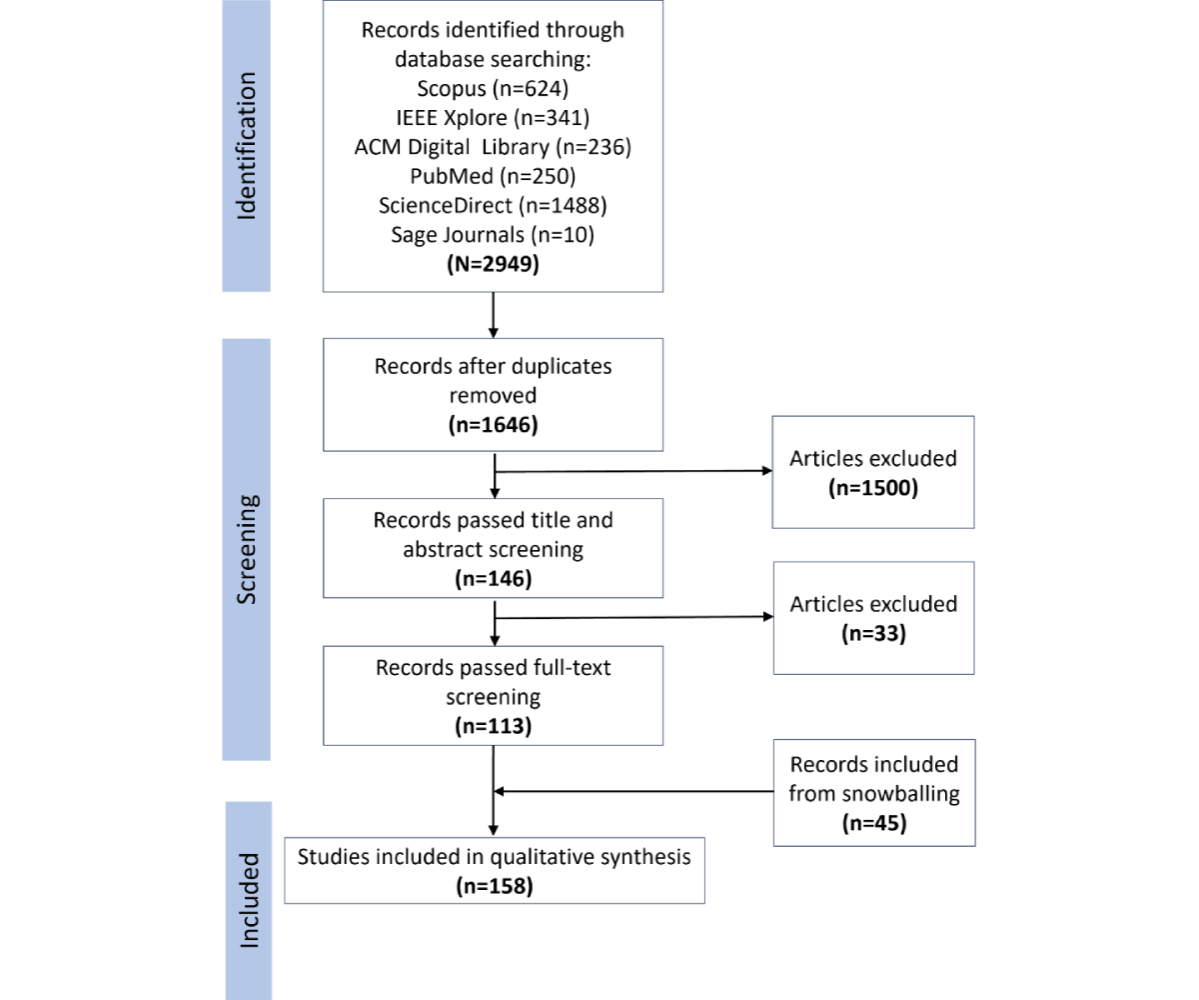
Study selection flowchart.

The study selection flowchart is presented in Figure 3. A total of 2949 articles were identified (n=2306, 78.2% retrieved through search strategy C and n=643, 21.8% retrieved through search strategy D). After screening and snowballing, 158 articles were included in this study (n=84, 53.2% from strategy C, n=29, 18.4% from strategy D, and n=45, 28.5% from snowballing). Of the 33 articles excluded after title and abstract screening, 7 (21%) were left out because they were in non-English languages.

### Subsections

**Figure 4:**
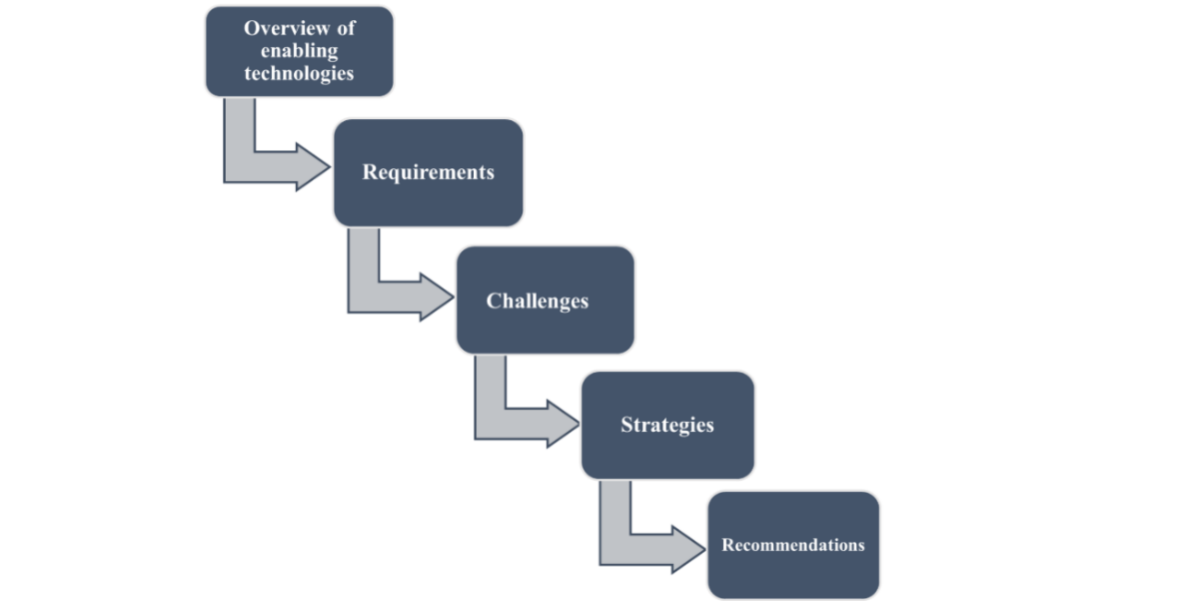
The main Results section is divided into 5 parts: overview (a summary of the enabling technologies that address various levels of interoperability), requirements (mapping Internet of Medical Things [IoMT] platform needs to the enabling technologies), challenges (presenting common interoperability challenges), strategies (outlining strategies to overcome these challenges), and recommendations (offering our research group’s suggestions for addressing interoperability issues in IoMT platform development).

This section is divided into 5 subsections (Figure 4). In the first, we provide an overview of the technologies and standards applicable to IoMT, emphasizing their relevance in addressing various levels of interoperability, as introduced in the research protocol [11]. In the second subsection, we present common interoperability requirements identified in the included studies, focusing on their application in prehospital care and HBC scenarios. Next, in the third subsection, we explore the interoperability challenges associated with IoMT platform development. In the fourth subsection, we summarize previous efforts in IoMT platform development and the technologies and standards used in these initiatives. Finally, in the fifth subsection, we draw upon our research findings to present actionable insights and recommendations for addressing interoperability challenges.

### An Overview of the Enabling Technologies That Address Interoperability Issues Within IoMT

In the following subsections, we have categorized and summarized the enabling technologies based on the specific interoperability level they address (refer to Multimedia Appendix 2 for a description of the enabling technologies).

#### Device Interoperability

Device interoperability is the foundational level of interoperability [36] and involves ensuring that heterogeneous devices can physically connect and communicate in a restricted network, typically a personal area network (PAN) [36]. Solving issues at this level is often the first step in IoMT platform development processes because interoperable sensors are a prerequisite for subsequent analysis and decision-making processes. However, the widespread use of vendors’ proprietary solutions often hinders the achievement of this level of interoperability [37]. Consequently, patients receiving hypertension management services from a particular company, for example, are often required to use a blood pressure device provided by the same company [38].

A variety of standards and protocols, including the Institute of Electrical and Electronics Engineers (IEEE) 11073 personal health device (PHD) [38-40], Zigbee [41], and Bluetooth Low Energy (BLE) [42], play a crucial role in addressing interoperability among medical devices, such as weighing scales, blood pressure monitors, and blood glucose monitors [39,43]. These technologies and devices can often be mapped to the perception layer in the IoMT reference model [44], a layer that is responsible for gathering raw data [45].

#### Network Interoperability

Network interoperability focuses on information exchange over the internet, including the ability of different networks or devices on separate networks to communicate [46]. Typically, network interoperability involves extended communication compared to device interoperability, which includes communication within local area networks (LANs) or wide area networks (WANs).

Technologies that focus on solving issues at the network interoperability level can primarily be mapped to the transport or processing layer in the IoMT reference model and include IP [47], user datagram protocol [48], transmission control protocol, IPv6 over low-power wireless PAN (6LoWPAN) [48,49], software-defined networking [36,50], gateways [51-53], message queuing telemetry transport (MQTT) [54], and WebSocket [55].

#### Syntactic interoperability

Syntactic interoperability, the third level of interoperability, involves data formats and data structures [36]. In the health care sector, both unstructured (eg, images, audio, and video streams) and structured data are used, which means that technologies used to address syntactic interoperability must be able to process diverse data types. Without syntactic interoperability, data might be sent to a system that is unable to process and use the information [56].

Technologies that focus on syntactic interoperability issues can be mapped to the processing layer in the IoMT reference model and include JSON [57], Health Level 7 version 2 (HL7v2) [58], and XML [59].

#### Semantic Interoperability

**Figure 5:**
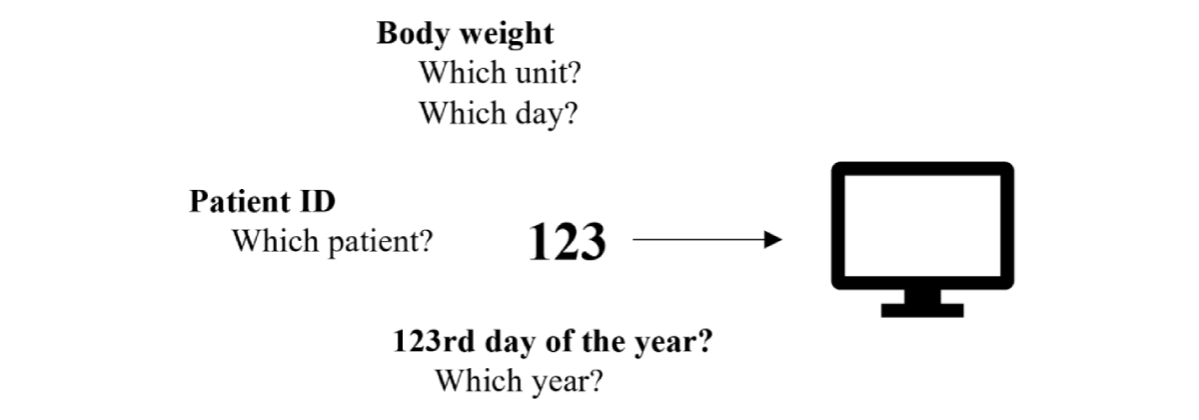
An example of the lack of semantic interoperability. If a system receives insufficient data, it cannot interpret the information correctly. In the absence of semantic interoperability between the sending and receiving systems and without additional context or information, the receiving system cannot interpret the value “123”.

Semantic interoperability refers to the ability of different computer systems to have a common understanding of message contents, enabling them to share data with unambiguous, shared meaning [25]. This level of interoperability is essential for enabling automatic data processing and decision-making in IoMT settings [60]. Shared semantics within health care can help to avoid knowledge mismanagement, clinical misinterpretation, misdiagnosis of a patient’s illness, and even patient deaths [61]; for example, a system that receives “123” as input from another system (Figure 5) cannot interpret the data without additional information. For the receiving system to process and use the received data, the value “123” must be complemented by relevant metadata tags, such as “systolic blood pressure” or “patient ID.” To achieve this contextual enrichment, well-established standards such as RxNorm [62], openEHR [63], *ICD* [37], Logical Observation Identifiers Names and Codes (LOINC) [62,64], and SNOMED CT [62] can be used. These standards offer a structured framework for associating informative labels and classifications with data [62].

#### Cross-Platform Interoperability

Cross-platform interoperability denotes the ability of different platforms within a single domain to work together seamlessly. It could be various systems (eg, PSAPs, EHRs, mobile apps, and laboratory information systems) running on different platforms (web, mobile, desktop, etc) located in different hospital wards or hospitals. Cross-platform interoperability challenges emerge at this level primarily due to the presence of a wide range of operating systems (Windows, Android, Linux, iOS, etc), programming languages, data structures, architectures, and access methods for both entities and data (eg, application programming interfaces [APIs]) [36]. Technologies that focus on addressing these levels of interoperability issues can be mapped to the application layer in the IoMT reference model and include Fast Healthcare Interoperability Resources (FHIR) [65-68], representational state transfer APIs (REST APIs) [66], microservices [7,69], Docker [70], Kubernetes [70], and cloud services [67,69-72].

#### Cross-Domain Interoperability

Patients are likely to receive medical attention from several institutions and across various domains over their lifetime. Hence, communicating vital information across organizational and national boundaries is essential to ensure proper patient care and treatment [29]. This level of communication is enabled by cross-domain interoperability, which represents the highest level of interoperability [36].

**Figure 6:**
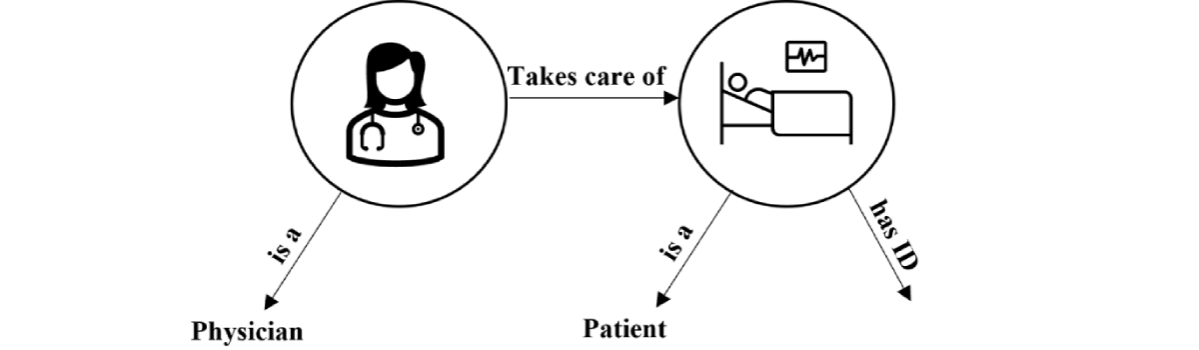
Ontologies define domain knowledge, and semantics provide data meaning. Semantics can tag data (eg, “systolic”), while ontologies provide context, such as relating “123” to “blood pressure” and defining “elevated systolic level” within the domain.

**Figure 7:**
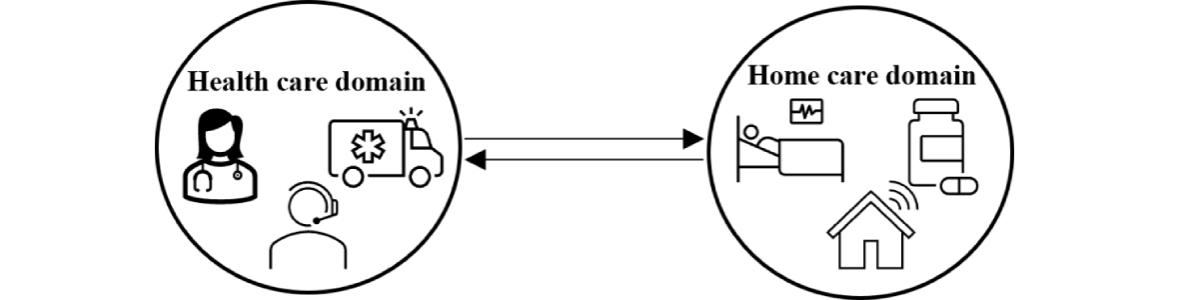
Technologies and standards such as blockchain, the General Data Protection Regulation (GDPR), and ontologies can enable data sharing between 2 separate domains.

In this context, a domain refers to a sociotechnical system defined by shared objectives and interests. These domains, or systems, are often separable from other systems by social, technical, and legal boundaries (Figures 6 and 7). Examples of domains include a medical device manufacturing company or a hospital. Each domain might have distinct goals, processes, security policies, and terminologies. Technologies and standards such as BioPortal [73], blockchain [74], ontology mediation [13,75], Web Ontology Language (OWL) [13], ontologies [25,74,76], General Data Protection Regulation (GDPR) [77,78], and Health Insurance Portability and Accountability Act (HIPAA) [78,79] can help achieve cross-domain interoperability.

### Common IoMT Interoperability Requirements for Prehospital Care and HBC

IoMT platforms for prehospital care and HBC have specific requirements. These platforms are required to operate in time-critical environments, where large amounts of sensitive data need to be exchanged in real time across various domains. In this subsection, we have summarized common interoperability requirements identified in the included studies and grouped them based on their potential application in IoMT settings (Table 1).

**Table 1:** The enabling technologies for interoperability and their application in Internet of Medical Things (IoMT) for prehospital care and home-based care. Suitable technologies are mapped to various IoMT platform requirements and potential use, with supporting references provided in the last column.

IoMT platform requirement	Potential use	Suitable technologies	References
Real-time text communication	Alerts, notifications, chats, and data visualization	MQTT^a^, WebSocket, and webhooks	[19,54,80-88]
Real-time videoconferences or audioconferences	Video and voice calls between patients and caregiver	WebRTC^b^ and VOIP^c^	[83,89]
Common data formats	Data interoperability and sharing	JSON, XML, CSV, and SenML^d^	[7,23,24,60,77,80,83,84,87,90,91]
Common semantics	Automatic data exchange, processing, and interpretation; persistent data storage and enabling use of AI^e^	FHIR^f^, LOINC^g^, SNOMED CT^h^, RxNorm, openEHR, ontologies, BioPortal, DMTO^i^, and HL7 CDA^j^	[19,23,24,60,77,82,88,92-94]
Data privacy and security	Encrypted data communication, access controls, and consent management services	Blockchain, HTTPS, TLS^k^, SSL^l^, GDPR^m^, and HIPAA^n^	[7,24,54,77,79,82-85,87,89,92,95,96]
Extended sensor communication	Allowing devices in LAN^o^ or PAN^p^ to send their data over internet (WAN^q^; eg, allowing monitoring applications outdoors)	Gateways, LPWAN^r^ (LoRa^s^ or LoRaWAN), and 3G, 4G, or 5G	[19,24,60,80,83,91,95,97-99]
System-to-system communication	Allowing systems located on different networks to connect over WANs	HTTP, REST API^t^, API, AMQP^u^, and SOAP^v^	[7,19,23,24,60,77,79,80,82-89, 92,94,96,100-102]
Allowing sensors from different vendors to communicate	Allowing sensors from different vendors to adhere to common data format and semantics	OneM2M and IEEE^w^ 11073	[19,24,94]
Translation or transcoding between ontologies in different domains or creation of common ontologies	Interpreting and processing information coming from a separate domain (eg, using AI)	OWL^x^, RDF^y^, OM^z^, Semantic Web, and HeTOP^aa^	[23,24,60,82,83,99,100,102]
Timely data processing and immediate responses in emergency situations	Minimizing delays and information loss in latency-sensitive and real-time applications (eg, heart monitoring systems)	Fog and edge computing	[7,19,79,80,83,85,91,97,98]
Device-to-device communication in PANs or LANs	Creating a smaller network of integrated medical devices that can collect patients’ vital signs	Zigbee, BLE^ab^, Bluetooth, Z-Wave, 6LoWPAN^ac^, ANT+^ad^, NFC^ae^, CoAP^af^, Wi-Fi, RFID^ag^, and IEEE 11073 PHD^ah^	[7,19,23,24,80,83,84,89-91, 94,95,97,99,100,103]
Modular system	Enhancing scalability and flexibility in IoMT; ensures consistent deployment and efficient application management	Microservices, Docker, and Kubernetes	[7,77,79,80,83,85]
Data availability and processing power	Improving administration and availability of data; enabling large-scale storage, computationally intensive data processing and advanced analysis tasks	Cloud services (computing and storage, etc)	[7,19,23,24,77,79,80,83,87,88, 91,93,95,97,100]
Common infrastructures or middleware	Platforms, frameworks, and infrastructure for creating interoperable, standardized, and scalable solutions	FIWARE, IHE^ai^, Continua, and OpenICE^aj^	[54,80,94]

^a^MQTT: message queuing telemetry transport.

^b^WebRTC: Web Real-Time Communication.

^c^VOIP: voice over IP.

^d^SenML: sensor markup language.

^e^AI: artificial intelligence.

^f^FHIR: Fast Healthcare Interoperability Resources.

^g^LOINC: Logical Observation Identifiers Names and Codes.

^h^SNOMED CT: Systematized Nomenclature of Medicine–Clinical Terms.

^i^DMTO: Diabetes Mellitus Treatment Ontology.

^j^HL7 CDA: Health Level 7 clinical document architecture.

^k^TLS: transport layer security.

^l^SSL: secure sockets layer.

^m^GDPR: General Data Protection Regulation.

^n^HIPAA: Health Insurance Portability and Accountability Act.

^o^LAN: local area network.

^p^PAN: personal area network.

^q^WAN: wide area network.

^r^LPWAN: low-power wide area network.

^s^LoRa: long range.

^t^REST API: representational state transfer application programming interface.

^u^AMQP: advanced message queuing protocol.

^v^SOAP: simple object access protocol.

^w^IEEE: Institute of Electrical and Electronics Engineers.

^x^OWL: Web Ontology Language.

^y^RDF: resource description framework.

^z^OM: ontology for units of measure.

^aa^HeTOP: Health Terminology/Ontology Portal.

^ab^BLE: Bluetooth Low Energy.

^ac^6LoWPAN: IPv6 over low-power wireless personal area network.

^ad^ANT+: Advanced and Adaptive Network Technology+.

^ae^NFC: near field communication.

^af^CoAP: constrained application protocol.

^ag^RFID: radio frequency identification.

^ah^PHD: personal health device.

^ai^IHE: Integrating the Healthcare Enterprise.

^aj^OpenICE: Open Integrated Clinical Environment.

### Common Interoperability Challenges in IoMT

Achieving interoperability is not a trivial task. The higher the interoperability level, the more complex the endeavor due to the involvement of additional technologies and standards. Previous research has reported recurring interoperability challenges frequently encountered in IoMT platform development for prehospital care and HBC. Some of these challenges are reported in Table 2.

**Table 2:** Common interoperability challenges in Internet of Medical Things (IoMT) platform development for prehospital care and home-based care. Each challenge is mapped to studies (references) that discuss it in the context of previous IoMT platform developments efforts.

Challenges	References
Latency	[11,13,22,75,79,81-85,91,101]
Privacy or security	[11,13,24,60,79,82,83,85,91,101-103]
Volume and complexity of data	[11,22,75,82-84,91,94,97,98,100,101,104]
Multiple or proprietary protocols	[11,56,60,79,83,85,91,98]
Different terminologies or semantics	[19,23,24,60,82,88,100,102]
Poor connectivity	[84,95,97,103]

Among the examined studies (n=25), we could identify 7 frequently reported challenges associated with interoperability. Among the 7 reported challenges, 12 (21%) were related to latency problems, 8 (14%) to proprietary protocols, 13 (23%) to the volume and complexity of data, 12 (21%) to privacy and security concerns, 8 (14%) to semantic coding issues and 4 (7%) to poor connectivity.

### Common Strategies to Overcome Interoperability Issues in IoMT Platform Development

In this section, we provide an overview of the technologies and standards used frequently to address interoperability challenges in IoMT platform development for prehospital care and HBC (Table 3).

**Table 3:** Summary of Internet of Medical Things (IoMT) platform development for home-based care and prehospital care as presented in 30 (19%) of the 158 studies reviewed, highlighting each IoMT platform’s primary purpose and objective, publication year, and the technologies and standards used to address interoperability issues.

IoMT platform	Objective	Publication year	Technologies and standards
System for sleep monitoring [80]	Real-time, remote monitoring of patients with sleep apnea to support diagnosis and treatment	2018	Fog and cloud computing, MQTT^a^, Zigbee, BLE^b^, CoAP^c^, 6LoWPAN^d^, FIWARE, JSON, CSV, HDFS^e^, REST API^f^, gateways, microservices, and Docker
IoT^g^-based message broker system [18]	Individual patient vital signs monitoring in potential emergency or prehospital applications	2022	RFID^h^, MQTT, REST API, Wi-Fi, CSV, XML, PDF, and JPEG
Platform for management of chronic diseases [23]	Care plan management tool integrated with clinical decision support services, EHRs^i^, and sensors	2019	FHIR STU3^j^, HL7 CDA^k^, CSV, XML, SOAP^l^, JSON, SNOMED CT^m^, LOINC^n^ and WHO ATC^o^, local versions of *ICD-10*^p^, REST API, cloud computing, HeTOP^q^, and SMART^r^ on FHIR
RePulmo^s^ [54]	Open-source platform for secure and accurate remote pulmonary data monitoring	2019	MQTT, OpenICE^t^, TLS^u^, and API
SPIDEP^v^ [7]	Support the early diagnosis of infectious diseases in older people	2020	Microservices, Docker, Kubernetes, cloud and edge computing, REST API, JSON, gateways, HTTPS, TLS, Wi-Fi, and SMART on FHIR
Semantic IoMT platform for eHealth [60]	An interoperable IoMT platform	2020	openEHR, OWL^w^, ontologies, protocol converter, Bluetooth, Zigbee, Wi-Fi, SenML^x^, gateways, JSON, REST API, cloud computing, and Semantic Web
IoMT platform for aggregation, processing, and sharing [19]	A platform to cover the domain of health care, following widely adopted standards, enabling semantic interoperability, and considering a big data approach	2019	REST API; MQTT; CoAP; openEHR; FHIR; SenML; Wi-Fi; cloud, fog, and edge computing; OneM2M; gateways, and Wi-Fi
Platform for health care promotion and cardiovascular disease prevention [96]	Smart health care platform oriented to multiple point-of-care scenarios for health care promotion and cardiovascular disease prevention	2021	REST API, TCP^y^, JSON, XML, SOAP, Bluetooth, Wi-Fi, ANT+^z^, HTTPS, TLS, cloud computing, and Wi-Fi
SemPryv [84]	SemPryv supports REST APIs to consume and produce interoperable streams of health care data, following the HL7 FHIR standard and using the Pryv.io platform	2023	FHIR, ontologies, SNOMED CT, LOINC, BioPortal, REST API, WebSocket, webhooks, RxNorm, UCUM^aa^, Docker, GDPR^ab^, Kubernetes, and RDF^ac^
LoRaWAN^ad^-based NXTGeUH^ae^ [103]	System for vital signs monitoring and fall detection	2018	Zigbee, LoRaWAN, TCP/IP, Bluetooth, and gateways
Monitoring platform for patients with diabetes [90]	Support treatment, monitoring, and data collection	2021	API, Bluetooth, JSON, and NFC^af^
VITASENIOR-MT [83]	Remote monitoring of health parameters of older people	2019	Cloud and fog computing, microservices, CoAP, JSON, BLE, WebSocket, REST API, MQTT, gateways, 6LoWPAN, AMQP^ag^, Bluetooth, WebRTC^ah^, GDPR, and HTTPS
Apnea MedAssist 2 [104]	Monitoring for Alzheimer disease diagnosis and rehabilitation	2013	Bluetooth, Wi-Fi, cloud computing, and 4G
IC-SMART^ai^ [100]	Monitoring for Alzheimer disease diagnosis and rehabilitation	2019	OWL, ontologies, gateways, REST API, RFID, Wi-Fi, Bluetooth, cloud computing, Docker, and HTTP
HealthFog [101]	Automatic diagnosis of heart diseases	2020	REST API; cloud, edge, and fog computing; gateways; CSV; blockchain; and HTTP
HABITAT^aj^ [102]	Platform for independent older people	2019	RFID, HTTPS, WebSocket, RDF, JSON, Semantic Web, ontologies, and SPARQL^ak^
SensorHub [84]	A central platform that acts as a bridge between sensors and a user’s smartphone	2022	BLE, Bluetooth, REST API, HTTPS, WebSocket, CSV, JSON, and Docker
Energy-aware IoT-based architecture [85]	On-wrist fall detection system	2023	MQTT, HTTPS, Wi-Fi, Docker, and API
Real-time remote monitoring system [91]	Low-cost health system for continuous monitoring of ECG^al^	2017	Bluetooth, Wi-Fi, gateways, cloud and fog computing, JSON, XML, API, and TCP
Fall detection system [97]	A system that can be used to monitor, for example, cardiovascular diseases or diabetes	2019	LoRa; edge, fog, and cloud computing; BLE; and gateways
ChainSDI [79]	Securing health care applications at home	2020	Edge and cloud computing, blockchain, Wi-Fi, Kubernetes, Docker, HIPAA^am^, HTTP, and API
SmartHabits [77]	A home care assistant system to support informal caregivers caring for persons living alone	2019	REST API, JSON, AMQP, gateways, cloud computing, ontologies, Z-Wave, HTTP, microservices, GDPR, and Wi-Fi
DALÍ [87]	A personal assistant platform for older people and those who are visually impaired	2020	JSON, MQTT, HTTPS, REST API, cloud computing, WebSocket, and SSL^an^
Integrated health monitoring system [94]	Integrated monitoring system	2015	FHIR, REST API, IEEE^ao^ 11073, Zigbee, Bluetooth, IHE^ap^, Continua, SOAP, JSON, and XML
A mobile health monitoring-and-treatment system [24]	Platform for diabetes monitoring and to provide customized, long-term, and real-time treatment plans	2019	FHIR, ontologies, OWL, JSON, HTTPS, REST API, Bluetooth, cloud computing, SNOMED CT, LOINC, RxNorm, BioPortal, DMTO^aq^, IEEE 11073, Wi-Fi, 3G, 4G, or 5G, and BFO^ar^
Patient monitoring system in smart homes [98]	Remote monitoring platform for monitoring patients in smart homes	2018	Cloud and fog computing, RFID, gateways, and Wi-Fi
VitalCore [88]	Allowing patients access to their health data in real time	2021	REST API, WebSocket, and HL7 data
Health care system for older people [99]	An ontology-based IoMT platform to alleviate problems related to chronic diseases	2021	Ontologies, gateways, Wi-Fi, Zigbee, LoRaWAN, RDF, and OWL
We-Care [95]	Platform to assist older people in their homes and trigger alarms in case of emergency situations; works offline	2017	Bluetooth, 6LoWPAN, gateways, UDP, Wi-Fi, cloud computing, and API
Remote pain monitoring system [81]	A scalable IoT system for real-time automatic pain assessment using facial expressions	2018	Gateways, UDP, TCP, Wi-Fi, WebSocket, TLS, and cloud computing

^a^MQTT: message queuing telemetry transport.

^b^BLE: Bluetooth Low Energy.

^c^CoAP: constrained application protocol.

^d^6LoWPAN: IPv6 over low-power wireless personal area network.

^e^HDFS: Hadoop Distributed File System.

^f^REST API: representational state transfer application programming interface.

^g^IoT: Internet of Things.

^h^RFID: radio frequency identification.

^i^EHR: electronic health record.

^j^FHIR STU3: Fast Healthcare Interoperability Resources Standard for Trial Use, version 3.

^k^HL7 CDA: Health Level 7 clinical document architecture.

^l^SOAP: simple object access protocol.

^m^SNOMED CT: Systematized Nomenclature of Medicine–Clinical Terms.

^n^LOINC: Logical Observation Identifiers Names and Codes.

^o^WHO ATC: World Health Organization Anatomical Therapeutic Chemical.

^p^ICD-10: International Classification of Diseases, Tenth Revision.

^q^HeTOP: Health Terminology/Ontology Portal.

^r^SMART: Substitutable Medical Applications, Reusable Technologies.

^s^RePulmo: remote pulmonary monitoring system.

^t^OpenICE: Open Integrated Clinical Environment.

^u^TLS: transport layer security.

^v^SPIDEP: System for Prediagnosis and Telecare of Infectious Diseases in Elderly People.

^w^OWL: Web Ontology Language.

^x^SenML: sensor markup language.

^y^TCP: transmission control protocol.

^z^ANT+: Advanced and Adaptive Network Technology+.

^aa^UCUM: Unified Code for Units of Measure.

^ab^GDPR: General Data Protection Regulation.

^ac^RDF: resource description framework.

^ad^LoRaWAN: long-range wide area network.

^ae^NXTGeUH: Next Generation Ubiquitous Healthcare.

^af^NFC: near field communication.

^ag^AMQP: advanced message queuing protocol.

^ah^WebRTC: Web Real-Time Communication.

^ai^IC-SMART: Internet of Things Cloud-Enabled Seamless Monitoring for Alzheimer Diagnosis and Rehabilitation.

^aj^HABITAT: Home Assistance Based on the Internet of Things for the Autonomy of Everybody.

^ak^SPARQL: SPARQL Protocol and RDF Query Language.

^al^ECG: electrocardiography.

^am^HIPAA: Health Insurance Portability and Accountability Act.

^an^SSL: secure sockets layer.

^ao^IEEE: Institute of Electrical and Electronics Engineers.

^ap^IHE: Integrating the Healthcare Enterprise.

^aq^DMTO: Diabetes Mellitus Treatment Ontology.

^ar^BFO: Basic Formal Ontology.

^as^UDP: user datagram protocol.

**Figure 8:**
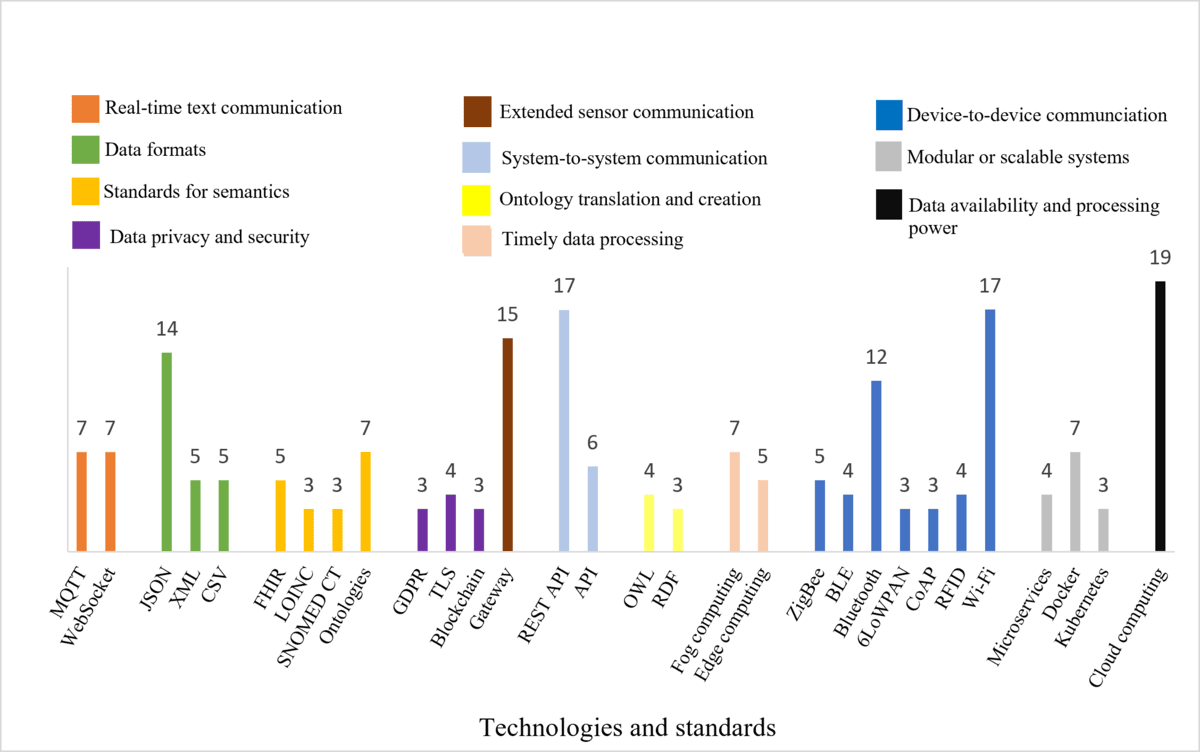
Occurrences of common technologies addressing interoperability in Internet of Medical Things (IoMT) platforms for prehospital care and home-based care, grouped by application area (each color). Only technologies and standards reported in 2 or more separate articles are included. 6LoWPAN: IPv6 over low-power wireless personal area network; API: application programming interface; BLE: Bluetooth Low Energy; CoAP: constrained application protocol; FHIR: Fast Healthcare Interoperability Resources; GDPR: General Data Protection Regulation; LOINC: Logical Observation Identifiers Names and Codes; MQTT: message queuing telemetry transport; OWL: Web Ontology Language; RDF: resource description framework; REST API: representational state transfer application programming interface; RFID: radio frequency identification; SNOMED CT: Systematized Nomenclature of Medicine–Clinical Terms; TLS: transport layer security.

As can be seen in Table 3, IoMT platform development often requires a combination of technologies and standards. Simply specifying “Bluetooth,” “Wi-Fi,” or “SNOMED CT” is not sufficient because no single standard, protocol, or technology can address higher levels of interoperability [100]; for example, SNOMED CT can be complemented by more domain-specific standards such as LOINC [29], while Bluetooth can be used in conjunction with a gateway, MQTT, and FHIR to enhance interoperability within IoMT [19]. Table 2 reveals variations in the frequency of the technology and standards used. Consequently, we have summarized the most used technologies and standards in Figure 8. Technologies and standards that appeared in 2 or fewer articles were excluded from Figure 8. These include Web Real-Time Communication (WebRTC), openEHR, voice over IP, webhooks, and Substitutable Medical Applications, Reusable Technologies (SMART) on FHIR, as well as the interoperability guidelines and frameworks such as FIWARE, Integrating the Healthcare Enterprise (IHE), Continua, and Open Integrated Clinical Environment (OpenICE). The top 5 reported technologies were cloud computing (19/37, 51%), REST APIs (17/37, 46%), Wi-Fi (17/37, 46%), gateways (15/37, 41%), and JSON (14/37, 38%).

### Recommendations

Our recommendations (Textbox 3) are based on the findings presented in Table 3 together with discussions within our research group. These recommendations focus on technologies and standards that support cross-domain interoperability across all layers of the IoMT reference model, spanning from data collection by sensors to cloud-based data processing and secure cross-domain data exchange. In broad terms, an IoMT platform for HBC and prehospital care should include, in our opinion, the capabilities presented in Textbox 3.

**Capabilities required by an Internet of Medical Things (IoMT) platform for home-based care and prehospital care.**

**Compatibility for medical device integration and secure and reliable real-time data transfer to the cloud**

- The IoMT platform should ensure seamless integration of medical devices and enable secure, real-time transmission of data to the cloud.
- Recommended technologies and standards: Bluetooth Low Energy (BLE), Zigbee, Institute of Electrical and Electronics Engineers (IEEE) 11073 personal health device (PHD), Open Integrated Clinical Environment (OpenICE), and FIWARE

**Real-time and low latency data processing and communication**

- The IoMT platform should be capable of processing and visualizing data in real time with minimal latency to support timely decision-making and data processing.
- Recommended technologies and standards: message queuing telemetry transport (MQTT), WebSocket, and Web Real-Time Communication (WebRTC)

**Data persistence for reliable data storage**

- The IoMT platform should ensure that data are stored in a consistent and secure way.
- Recommended technologies and standards: openEHR and blockchain

**Open data standards and data exchange mechanisms for secure and efficient system-to-system communication.**

- The IoMT platform should adhere to open data standards and secure exchange mechanisms, facilitating efficient communication between different systems.
- Recommended technologies and standards: JSON and representational state transfer application programming interface (REST API)
- Common semantics and domain knowledge descriptions for automatic knowledge extraction

**Common semantics and domain descriptions enable automatic extraction of knowledge from data, enhancing system understanding.**

- Recommended technologies and standards: Fast Healthcare Interoperability Resources (FHIR), Systematized Nomenclature of Medicine–Clinical Terms (SNOMED CT), Web Ontology Language (OWL), resource description framework (RDF), Logical Observation Identifiers Names and Codes (LOINC), and BioPortal

**Security measures to protect patient data and ensure data confidentiality**

- The IoMT platform should ensure the privacy and integrity of patient data throughout its life cycle.
- Recommended technologies and standards: transport layer security (TLS), General Data Protection Regulation (GDPR), Health Insurance Portability and Accountability Act (HIPAA), blockchain, and Open Authorization 2.0 (OAuth2)

**Scalability to accommodate a growing number of connected devices and generated data volumes**

- The platform should scale effectively to handle an increasing number of devices and growing data volumes.
- Recommended technologies and standards: Docker, Kubernetes, cloud services, and fog and edge computing

## Discussion

### Principal Findings and Study Contribution

This scoping review provides insights into the enabling technologies that can address interoperability issues in IoMT settings. Although the primary focus has been on solutions for prehospital care and HBC, the results are also applicable to other settings with similar characteristics and requirements. The results show that higher levels of interoperability in IoMT can be achieved by combining various technologies and standards from multiple interoperability levels.

On the basis of the studies (n=30) presented in Table 3, it seems that contemporary IoMT studies on prehospital care and HBC tend to focus on lower levels of interoperability. This conclusion is in line with previous research conducted by Rubí and Gondim [19]. One possible explanation for the predominant focus on device integration and lower levels of interoperability among the reviewed studies is our use of “IoMT” as a search term. Although IoMT is broad and includes integration with medical devices as well as medical applications, the term is somewhat ambiguous and might introduce some biases toward device-centric approaches. We believe that future research could benefit from using a more refined definition to emphasize a shifted focus toward higher levels of interoperability. Therefore, we propose the introduction of the term Internet of Medical Systems (IoMS) to address this gap. This new terminology could help distinguish studies focusing on device-to-device integration from those focusing on integration with EHRs and other health applications.

We acknowledge that some may argue that technologies and standards such as blockchain, fog computing, GDPR, and the like may primarily focus on aspects other than interoperability (eg, security, communication, networking, data transfer, or architectural design). However, proceeding from the 6-level interoperability model [11], our interpretation of interoperability extends beyond mere data exchange and interpretation. Even if two systems possess the capabilities to exchange and interpret information, we believe that it is not enough to achieve higher levels of interoperability within health care without considering privacy and security aspects. However, our findings indicate varying levels of focus on technologies such as blockchain and SMART on FHIR within the research community, with some of the studies (4/30, 13%) analyzing these technologies in depth, while others (26/30, 87%) tend to overlook them. As an example, of the 30 included studies, 8 (27%) reported the use of authentication and authorization mechanisms [79,80,90,91,95,96,101,105], but only 7% (2/30) reported the use of Open Authorization (OAuth) or SMART on FHIR as mechanisms for authentication and authorization [7,23].

On the basis of our findings, it seems that more comprehensive platforms tend to incorporate a wider range of technologies compared to simpler platforms (Table 3); for example, platforms that reported interoperability capabilities with external systems or the use of AI seem to more frequently use technologies and standards to semantically code the data, including ontologies, FHIR, LOINC, *ICD*, openEHR, RxNorm, and SNOMED CT. However, this relationship was not confirmed statistically, and we acknowledge that other factors may influence platform complexity, such as regulatory requirements, available technologies, and expertise among developers.

Table 3 reveals that 30% (9/30) of the IoMT platform development studies for prehospital care and HBC reported the use of semantic frameworks or ontologies. As the creation of ontologies is a necessary but time-consuming and labor-intensive task, research has examined the potential to achieve ontology alignment by using AI; for example, Dam et al [106] used augmented intelligence to map data across various domains to facilitate the integration of diverse data sources. According to Tangi et al [107], AI has the potential to not only establish a common language and foster a shared understanding of data but also clean and structure the data. While we acknowledge that using AI for interoperability is an emerging trend, such studies have not been incorporated in this review.

In the reviewed literature, semantic standards and ontologies were described as essential for establishing connections with external systems and enabling the use of AI. Interestingly, standards for image and video formats, such as Digital Imaging and Communications in Medicine (DICOM), have not been mentioned in the examined studies. Interoperability enhancing organizations and initiatives such as FIWARE, IHE, Continua, and OpenICE were reported to guide researchers and developers [88], but these organizations and initiatives were only mentioned in 3 (10%) of the 30 studies.

Fog and edge computing were reported to be used to overcome problems related to limited bandwidth, often associated with high data rate applications, such as fall detection systems or electrocardiography monitoring [97]. Our findings suggest that fog and edge computing are often combined with cloud computing, especially in AI-intensive applications that require more processing power. To improve the performance of AI algorithms and enable machines to correlate data, studies reported the use of ontologies, resource description framework (RDF), and OWL [24,99]. Furthermore, platforms requiring real-time functionalities (alerts or notifications) reported the use of MQTT (7/30, 23%) and WebSocket (7/30, 23%) [82,85].

While we have documented the use of commonly used technologies and standards in this study, it is important to acknowledge that the accuracy of these findings depends on the transparency and thoroughness of the explanations within the included studies; for instance, some of the studies (30/63, 48%) offered detailed insights into their IoMT platform development processes and the technologies and standards used, while others (33/63, 52%) focused on broad aspects of the topic, as in the studies by Rakhman et al [108] and Shim et al [109]. As a result, some of the studies (33/63, 52%) were excluded from Table 2. Furthermore, because technologies and standards can have versatile applications, specifying their exact application area can often be challenging. Therefore, the findings presented in Table 3 should be considered as a guidance rather than absolute truths.

### Mapping the Enabling Technologies to the Interoperability Model

In this study, the mapping was performed based on a 6-level interoperability model defined in the study protocol [11]. To facilitate the mapping process, any inconsistencies between our model and the levels described in the included studies were addressed using a flowchart model [11]. This was done to manage different naming conventions across different models; for instance, one study may label a specific interoperability level as “technical interoperability,” [105] while others may refer to it as “foundational interoperability” [110]. Similarly, the specific functions and components of each level may not be consistent across different models or frameworks, which can create ambiguity and uncertainty about how to define and interpret interoperability. Hence, proceeding from a different interoperability model [71] would most likely result in a somewhat different mapping outcome.

It is important to note that although we mapped the enabling technologies to specific interoperability levels, it is uncommon for a technology to address a single interoperability level; for example, the FHIR standard not only specifies data exchange formats and semantics but also supports REST APIs, enabling it to address syntactic, semantic, and cross-platform interoperability concurrently. Hence, in this study, we mapped FHIR to cross-platform interoperability, which represents the highest level of interoperability addressed by FHIR. However, saying that the FHIR standard *solves* cross-platform interoperability is not completely true because it needs to be combined with technologies on lower interoperability levels. We argue that the correct phrasing should be that FHIR *can* solve cross-platform interoperability issues if it is appropriately combined with other relevant technologies and standards.

Another aspect worth mentioning regarding the mapping is the importance of examining the primary focus of the technologies and standards; for example, the IEEE 11073 PHD standard involves data models that specify how a measurement and observations of vital signs should be represented, including descriptions of the different units, data types, and semantic meanings [38,40]. On the basis of this description, it is likely that the IEEE 11073 PHD standard should be categorized at the semantic interoperability level. However, due to its primary focus on device-to-device communication within LANs or PANs, the IEEE 11073 PHD standard does not fully meet the requirements for network interoperability, let alone semantic interoperability. Hence, the IEEE 11073 PHD standard was mapped to the device interoperability level in this study.

### Summarizing the Enabling Technologies That Address Interoperability Issues

In our study, we found that technologies such as BLE and Zigbee, which primarily target device interoperability, are not sufficient for IoMT applications in prehospital or HBC settings due to their restricted physical communication range (ie, 20-100 meters) [41,111]. For physicians to remotely monitor patients and for devices to automatically send alarms during medical emergencies, the first step is to extend the communication range by enabling network interoperability [49]. Otherwise, the physician’s system would need to be in the same room as the patient, which obviates the concept of remote monitoring; for example, by combining BLE with long-range wide area network (LoRaWAN) or 4G or 5G—or 6LoWPAN with CoAP—long-range data transmission can be achieved [112]. This allows physicians to remotely monitor patients residing several kilometers away from the hospital [25]. However, choosing a particular combination of technologies from different interoperability levels does not ensure the achievement of the desired level of interoperability. This is because interoperability initiatives require collaboration among all involved stakeholders; for instance, a standard such as IEEE 11073 can be adopted in several ways, which means that simply specifying the use of IEEE 11073 may not be sufficient to achieve device interoperability. Instead, a standard should be tailored to meet the specific requirements of a particular use case or application. This can be achieved using profiles to ensure that the standard is implemented in a way that suits the needs.

Although close collaboration among stakeholders is essential to achieve higher levels of interoperability, studies have shown this endeavor to be challenging; for example, a study conducted in 2021 by Everson et al [113] revealed that more than half (55%) of health information exchange organizations in the United States reported varying degrees of intentional information blocking. The authors showed that the most prevalent form of information blocking involved the refusal to share information, which 14% of the health information exchange organizations routinely observed among EHR vendors. The reason for blocking information according to Everson et al [113] had to do with regional competition among vendors. By limiting the sharing of information with other actors, vendors can ensure that health care providers stick to their platform [113,114]. While this approach benefits vendors, it creates disadvantages for patients and the health care industry. The findings of Everson et al [113] suggest that while technologies and standards can streamline data exchange in health care, the reluctance of organizations to engage in such efforts impedes progress toward higher levels of interoperability.

### Comments on Our Recommendations

The recommendations provided in this study draw on insights from previous IoMT platform development projects and our research group’s expertise. They focus on technologies and standards that together can support cross-platform or cross-domain interoperability. We acknowledge that multiple technologies and standards can be used to develop IoMT platforms. We are also aware that there is no “one right way” of adopting a specific technology or standard. Rather, we argue that interoperability initiatives and development projects should be facilitated by close collaboration among the stakeholders involved.

With this study and our recommendations, we hope to bring together the research, developer, and health care communities by highlighting relevant technologies and standards to foster collaboration and improve interoperability initiatives within the health care domain. While we recognize the importance of independence in driving innovation, we hope to see more IoMT platforms using common standards and best practices already available in the market. We further hope to see an increased engagement in interoperability initiatives such as FIWARE, IHE, and OpenICE.

We acknowledge the advantages of IoMT platforms capable of delivering personalized patient care and enhancing patient safety. However, we do not believe that technologies and standards are the only solutions. To achieve the highest level of interoperability, we argue that it is necessary to have a profound understanding of organizations’ working processes, routines, and policies. Hence, we look forward to delving into these challenges in close collaboration with relevant stakeholders and developing an IoMS platform that supports cross-domain interoperability.

### Limitations and Biases

This scoping review adheres to the published protocol [11] and focuses on technologies addressing interoperability issues within the context of IoMT platform development in HBC and prehospital care settings. Hence, studies focusing on hospital systems [115], platforms for intensive care units [81], integration platforms [93], or platforms for research or clinical trials [92,116] were omitted. Furthermore, most of the studies (33/63, 52%) displayed a deficiency in information or a lack of transparency regarding the development process and the technologies used; therefore, they were excluded from Table 1 and Table 3. As the focus of this study was to identify and summarize best practices and frequently used technologies, we did not assess the effectiveness of technology implementations in detail.

To manage the scope of this review process effectively, the reviewers made a deliberate decision to limit the included studies to those specifically addressing interoperability in an IoMT context. This was necessary to avoid overwhelming screening efforts with an unmanageable number of studies. We are aware that we might have missed relevant articles with this strategy. Furthermore, we are aware that technologies used in other sectors, for example, Industry 4.0, manufacturing, and transportation, could also be applicable to IoMT, especially considering that these domains share some common characteristics (eg, requirements for real-time, cross-domain information exchange). Another limitation of this study might be the use of the term “IoMT” in our literature search. Although IoMT is often defined as the interconnection of medical devices and applications, there seems to be a predominant focus on devices and lower levels of interoperability among these studies. This limitation could potentially be eliminated by the introduction and use of the new term IoMS.

Although recent advancements in AI have demonstrated its potential to enhance interoperability, studies reporting the use of AI for this purpose were excluded from this review. Another limitation of this study is that, to expedite the process, the coding scheme was tested by only 1 reviewer (MS).

### Conclusions

In this study, we have demonstrated that the highest level of interoperability can be theoretically achieved through a strategic combination of various technologies and standards. Furthermore, we have provided a summary of the relevant technologies and standards that can be used in IoMT platforms to overcome interoperability issues in HBC and prehospital care settings.

Despite the availability of innovative and suitable technologies, the IoMT research community has reported limited interest in adopting technologies and standards such as Docker, blockchain, SMART on FHIR, HIPAA, and GDPR to achieve cross-domain interoperability. Most of the studies (17/30, 57%) have primarily focused on lower levels of interoperability (up to the semantic interoperability level). This observation highlights a significant research gap, particularly in achieving cross-domain interoperability within IoMT for HBC and prehospital care. To emphasize the need for higher levels of interoperability and to support future research, we advocate for the introduction of the term IoMS. In addition, the reluctance of organizations and vendors to share information and participate in interoperability initiatives highlights the importance of considering these aspects when addressing interoperability challenges, especially in procurement processes [117].

## Appendix

Multimedia Appendix 1 PRISMA-ScR (Preferred Reporting Items for Systematic Reviews and Meta-Analyses extension for Scoping Reviews) checklist.

Multimedia Appendix 2 Search strategies for electronic databases and mapping result.

## Abbreviations

6LoWPAN: IPv6 over low-power wireless personal area network
API: application programming interface
BLE: Bluetooth Low Energy
DICOM: Digital Imaging and Communications in Medicine
EHR: electronic health record
FHIR: Fast Healthcare Interoperability Resources
GDPR: General Data Protection Regulation
HBC: home-based care
HIPAA: Health Insurance Portability and Accountability Act
HL7v2: Health Level 7 version 2
ICD-10-CM: International Classification of Diseases, Tenth Revision, Clinical Modification
IEEE: Institute of Electrical and Electronics Engineers
IHE: Integrating the Healthcare Enterprise
IoMS: Internet of Medical Systems
IoMT: Internet of Medical Things
ISO: International Organization for Standardization
LAN: local area network
LOINC: Logical Observation Identifiers Names and Codes
LoRaWAN: long-range wide area network
MQTT: message queuing telemetry transport
OAuth: Open Authorization
OpenICE: Open Integrated Clinical Environment
OWL: Web Ontology Language
PAN: personal area network
PHD: personal health device
PRISMA-ScR: Preferred Reporting Items for Systematic Reviews and Meta-Analyses extension for Scoping Reviews
PSAP: public safety answering point
RDF: resource description framework
REST API: representational state transfer application programming interface
SMART: Substitutable Medical Applications, Reusable Technologies
SNOMED CT: Systematized Nomenclature of Medicine–Clinical Terms
WAN: wide area network

## Data Availability

All datasets generated or analyzed during this study are included in this published paper and its supplementary information files.
